# Prevalence of SARS-CoV-2 Infection Among Vulnerable Populations Relying on Public Health Services: Findings from the AVISA Study in Brazil

**DOI:** 10.1590/0037-8682-0151-2025

**Published:** 2026-02-16

**Authors:** Eliana Nogueira Castro de Barros, Manuela de Almeida Roediger, Ricardo Queiroz Gurgel, Marco Antonio de Oliveira, Victor Santana Santos, Lorena Guadalupe Barberia

**Affiliations:** 1 Instituto Butantan, Centro de Ensaios Clínicos e Farmacovigilância, São Paulo, SP, Brasil.; 2 Universidade Federal de Sergipe, Departamento de Medicina, Aracaju, SE, Brasil.; 3 Universidade Federal de Sergipe, Centro de Ensaios Clínicos, Laranjeiras, SE, Brasil.; 4 Universidade Federal de Sergipe, Programa de Pós-Graduação em Ciências da Saúde, Aracaju, SE, Brasil.; 5 Universidade de São Paulo, Faculdade de Filosofia, Ciências Humanas e Letras, Departamento de Ciência Política, São Paulo, SP, Brasil.

**Keywords:** SARS-CoV-2 infection prevalence, Social inequity, Vulnerability, Brazilian Family Health Strategy

## Abstract

**Background:**

Understanding SARS-CoV-2 seroprevalence among vulnerable populations is crucial, especially in countries with universal but resource-constrained healthcare systems, such as Brazil.

**Methods:**

This was a cross-sectional, population-based household survey within the AVISA study, which aimed to assess seroprevalence among households enrolled in the Family Health Program (ESF). Two serological assays (rapid test and ECLIA) were used to detect anti-SARS-CoV-2 antibodies. Descriptive statistics were calculated and survey-adjusted Poisson regression models with robust variance were estimated, yielding prevalence ratios (PRs) and 95% confidence intervals (CIs).

**Results:**

The overall weighted seroprevalence was 35.66% (95% CI: 30.55-40.77) in 2,986 participants. Compared with individuals in the Southeast region, participants in the Northeast and North regions had a 1.48- and 1.38-times higher prevalence of SARS-CoV-2 testing positivity, respectively. Relative to individuals who self-identified as having Black or Brown skin color using IBGE-defined categories, White had a 22% lower prevalence of seropositivity (PR = 0.78, 95% CI 0.63-0.97). Compared to those living in households with four to five family members, individuals living in households with six or more family members had a higher prevalence of seropositivity (PR = 1.34, 95% CI 1.05-1.71). Individuals in the lowest per capita household income quintile were more likely to have SARS-CoV-2 infection (PR = 1.89, 95% CI 1.21-2.95) than those in the highest.

**Conclusion:**

This study is the first to assess SARS-CoV-2 seroprevalence among ESF participants, a socioeconomically vulnerable population, served by the primary care public health system. Lower socioeconomic status was associated with higher seroprevalence.

## INTRODUCTION

Coronavirus disease 2019 (COVID-19), a public health emergency of international concern (PHEIC), that began on January 30^th^, 2020^1^, has resulted in more than 776 million confirmed cases of COVID-19 worldwide and more than 7.1 million deaths^2^. Before vaccines became available, rigorous testing and non-pharmacological interventions (NPIs) were the most effective policies to reduce the spread^3^. 

However, low-income populations face significant challenges in adhering to social distancing during the COVID-19 pandemic. Studies have shown that the magnitude of the COVID-19 pandemic in Brazil can be partially explained by the country’s socioeconomic disparities^4^. In Brazil, official estimates show that poverty has reached its highest level since 2012, with 29.6% of the population living in poverty by 2021, and a 22.7% increase in the number of people living in poverty^5^.

Most seroepidemiological studies in Brazil have focused on the general population^6^ ^-^ ^8^. However, some studies have suggested that these patterns are distinct in resource-poor settings with higher seroprevalence ratios^9^. In analyses of three waves of national serological surveys in Brazil, the prevalence of antibodies against SARS-CoV-2 showed steep class and ethnic gradients, with the lowest risk among White, educated, and wealthy individuals. A high prevalence of COVID-19 has been reported in northern Brazil and Manaus, even after the first wave of the pandemic^10^. For example, Rio de Janeiro, a city of over six million inhabitants in Southeast Brazil, where approximately 22% of residents live in favelas (slums or communities) or informal settlements, was severely affected by the pandemic, and recorded one of the highest mortality rates in the country^11^. 

Vulnerable populations experience poverty, material insecurity, inappropriate housing conditions, and a lack of access to essential services, such as sanitation and clean water. They also relied on overcrowded public transportation. Few studies, particularly those from low- and middle-income countries, have examined this population segment, which is vulnerable and largely dependent on public health services. 

In this study, we aim to describe the baseline prevalence of SARS-CoV-2 infection, as measured by serological response, among children, adolescents, and adults enrolled in the Brazilian Family Health Strategy (*Estratégia Saúde da Família* - ESF) in 11 municipalities in Brazil. The results reported in the study are the baseline results for participants in the AVISA (*Avaliação de incidência de infecção por SARS-CoV-2 e de COVID-19 no Brasil*) study, which was a longitudinal, prospective, observational cohort study conducted between October 2020 and May 2022 to evaluate SARS-CoV-2 transmission dynamics. The study reports participants' initial demographic, clinical, and behavioral characteristics at enrollment in the AVISA study. Based on the per capita income data collected in the survey, these households were considered vulnerable because the mean per capita household income (R$646) was significantly below the national per capita income for Brazil (R$1,380). 

## METHODS

### Study Design and Sampling Method

The target population was individuals enrolled in the ESF, which is the primary healthcare system of the Unified Health System (*Sistema Único de Saúde- SUS*)^12^. Study sites from eleven municipalities in four Brazilian regions were selected: São José do Rio Preto (Sao Paulo [SP]), Serrana (SP), São Paulo (SP), Belo Horizonte (Minas Gerais [MG]), and Rio de Janeiro (Rio de Janeiro [RJ]) in the Southeast region; Laranjeiras (Serigipe [SE] and Fortaleza (Ceara [CE]) in the Northeast region; Brasília (Federal District [DF]) and Cuiabá (Mato Grosso [MT]) in the Central-West region and Boa Vista (Roraima [RR]) and Porto Velho (Rondonia [RO]) in the Northern region (Supplementary Material 1). The AVISA sites were selected because the study centers were pre-qualified to conduct clinical trials and were experienced in collaboration with the Butantan Institute for COVID-19 research. A collaborating research center from the South of Brazil was not included in the study.

The microarea and area of coverage defined by the ESF of each municipality were used for the two levels of the probabilistic sampling method to select potential participant households. The study population was stratified and estimated to be distributed representatively into nine age groups (0-9, 10-19, 20-29, 30-39, 40-49, 50-59, 60-69, 70-79, ≥80 years). All individuals in the sampled households were invited to participate until the estimated sample size by age group was reached. 

### Eligibility criteria

Eligible participants were individuals of any sex or age enrolled in the ESF at the participating study center who provided signed informed consent. For those without a legal capacity, a legal representative provided consent. Individuals who self-reported a prior history of SARS-CoV-2 infection or were vaccinated against COVID-19 were excluded.

### Data Collection

After informed consent was obtained, information on household sociodemographics, including income, access to publicly provided water and sewage, and number of individuals living in the household, was collected. Self-reported responses for comorbidities, NPI adherence (e.g., self-reported efforts to reduce trips outside the home and mask use), reliance on public transportation, and other risk factors related to the risk of infection during the previous four weeks were obtained from each participant based on tabulated responses to the data collected from a questionnaire administered by the health team visiting the household (Supplementary Material 1). 

A blood sample of all the participants was collected at the initial visit for a serological test to detect anti-SARS-CoV-2 antibodies by quantitative electrochemiluminescence ECLIA (Elecsys® anti-SARS-CoV2 S and N; Sensitivity 98.8% and Specificity 99.9%). Additionally, an IgG/IgM rapid serological test was performed (Humasis®; sensitivity 91.6% and specificity 86.5%). Both the tests were conducted during the same visit and were not sequential. Specimens were classified as positive if any of the following criteria were met: (i) when RBD or IGT results were available, a reactive result for either marker (RBD = reactive or IGT = reactive); or (ii) in the absence of both RBD and IGT results, a positive result for IgG or IgM. Specimens were classified as negative using the following complementary criteria: (i) when both RBD and IGT results were available, nonreactive results for both markers (RBD = nonreactive and IGT = nonreactive); or (ii) in the absence of RBD and IGT results, negative IgG and/or IgM results. For sensitivity, individuals without RBD or IGT results were excluded (see Supplementary Material 1).

### Ethical issues

The study was approved by the ethical review board of the Comitê de Ética do Hospital Leforte de São Paulo Ethics Committee (CAAE: 31228620.1.1001.548) and by the ethical boards from each study site. The study was conducted according to the principles of the Declaration of Helsinki. Before inclusion, all the participants or their legal representatives signed written informed consent forms. 

### Statistical analysis

The outcome of interest was seropositivity for SARS-CoV-2 infection at the initial visit, as detected by serological and/or rapid tests. Considering preexisting medical conditions and sociodemographic and household income, descriptive statistics were calculated using 95% confidence intervals (CIs). All estimates were obtained by considering the complex sampling design by age group at each participating center. 

The sample size calculation was based on the formula of Hanley et al.^13^, stratified into nine age groups according to decades of life, considering a type I error of 5% (two-tailed), an estimated risk of SARS-CoV-2 symptomatic infection of 10%, and hospitalization estimates from 50 years of age, as described by Verity et al.^14^. The final sample size (n=3,520), adjusted considering a 20% dropout rate, was divided by the study site (initially calculated for 11 sites), such that approximately 320 participants from each municipality were expected to be enrolled. No design effect was applied, and no adjustments were made for non-responses. Sampling weights were adjusted for post-stratification based on the characteristics of individuals enrolled in the ESF in each municipality, considering the municipality's micro-area and the target population's age group. 

Prevalence ratios (PR) were obtained using Poisson regression with survey-adjusted robust standard errors. A robust Poisson model was preferred because of its advantage in directly estimating PR or the ratio of infected individuals to the total population. The significance level was set at p < 0.05. The PR were adjusted for sex, age group, skin color, household size, region, and semester of the initial intake visit when the blood samples were obtained. The frequency of material and access to essential services, which were determined as vulnerability conditions at the individual and household levels, were compared between seropositive and seronegative individuals. 

The analyses were performed using Stata v.18.0 (StataCorp LLC, College Station, TX, USA) survey package for sample weights. The standard errors were adjusted using the svyset command. The complex survey design was achieved by declaring the municipality's micro-area as the primary sampling unit, applying the sampling probability weight, and incorporating stratification by study center to obtain design-based variance estimates.

## RESULTS

A total of 3,077 individuals were recruited based on the eligibility criteria, and 12 were excluded from initial screening. Two study sites did not reach the expected sample size: Brasília (88 participants out of 320 initially planned) and São Paulo (44 out of 320). Following the verification of the inclusion criteria for analysis, an additional 77 participants were excluded (44 from the São Paulo site, which was entirely excluded from the final analysis), resulting in a final study population of 2,988 (Supplementary Materials S3). Two participants were excluded because their baseline test results were unavailable. Therefore, the final study population included 2,986. These individuals belonged to a total of 840 households. 

The weighted seroprevalence was estimated to be 35.66% (95% confidence interval [CI]: 30.55-40.77) (Table 1). Compared with the Southeast region, participants residing in municipalities in the Northeast region had a 1.48-fold higher prevalence (95% CI 1.15-1.92), and those in the North had a 1.39-fold higher prevalence (95% CI 1.14-1.69) (Table 1). After adjusting for age, self-reported IBGE-defined skin color, sex, household size, and recruitment period, no statistically significant differences in prevalence were observed between participants in the Southeast and those in the Center-West (Table 1).

Baseline SARS-CoV-2 prevalence reflects the epidemiological context at the time that individuals entered the study. Enrollment in the study varied by site, and the time to complete enrollment of individuals averaged 80 days per site, with 68.92% of the patients enrolled by December 2020. The final patient was enrolled in October 2021. Compared to individuals enrolled in the second semester of 2020, participants enrolled in the first semester of 2021 had a lower prevalence of antibodies (PR = 0.66, 95% CI 0.51-0.86) (Table 1). In contrast, those who entered the study in the second semester of 2021 had a 1.99-fold higher prevalence (95% CI 1.15-3.46) (Table 1).

**TABLE 1:** Prevalence and prevalence ratios of SARS-CoV-2 by sociodemographic characteristics, AVISA**.**

Characteristic	Participants		Prevalence		Prevalence Ratio^a^		P-value^a^
	n	Weighted (%)	Weighted (%)	95% CI	Ratio	95% CI	
**Overall**	2,986	100	35.66	30.55-40.77			
**Sex**							
Male	1,226	41.07	34.46	28.50-40.41	0 .99	0 .84-1.16	
Female	1,760	58.93	36.50	30.81-42.20	1		0.90
**Age Group (years)**							
0-9	167	5.61	30.97	18.04-43.89	0.85	0.55-1.31	0.47
10-19	330	11.05	40.42	31.95-48.89	1.13	0.86-1.49	0.38
20-29	470	15.73	36.63	27.97-45.30	1		
30-39	382	12.83	41.23	33.19-49.29	1.08	0.85-1.39	0.52
40-49	438	14.67	35.30	25.30-45.32	0.98	0.72-1.33	0.89
50-59	464	15.53	40.65	31.53-49.77	1.15	0.83-1.60	0.40
60-69	404	13.52	28.02	21.27-34.77	0.79	0.58-1.09	0.16
70-79	192	6.41	23.03	14.14-31.93	0.69	0.46-1.04	0.08
80+	139	4.65	35.45	19.68-51.21	1.08	0.67-1.73	0.77
**Self-reported skin color/race using IBGE categories**							
Brown and Black	2,185	73.17	38.30	32.85-43.75	1		
White	762	25.53	27.90	21.31-34.49	0.78	0.63-0.97	0.03
Asian and Indigenous	39	1.29	39.76	2.56-76.96	1.02	0.49-2.10	0.96
**Size of Household**							
1-3 members	950	31.81	31.40	24.54-38.33	1.01	0.79-1.29	0.96
4-5 members	997	33.39	32.52	24.88-40.17	1		
6+ members	1,039	34.80	42.54	34.54-50.55	1.34	1.05-1.71	0.02
**Region**							
**Central-West**							
Cuiabá (MT) and Brasília (DF)	94	3.15	24.75	14.55-34.94	0.71	0.44-1.16	0.17
**North**							
Boa Vista (RR) and Porto Velho (RO)	234	7.84	50.17	43.51-56.82	1.39	1.14-1.69	<0.01
**Northeast**							
Fortaleza (CE) and Laranjeiras (SE)	738	24.70	55.52	44.91-66.14	1.48	1.15-1.92	< 0.01
**Southeast**							
Belo Horizonte (MG)							
Rio de Janeiro (RJ)	1,920	64.31	26.80	21.26-32.34	1		
São José do Rio Preto (SP) and Serrana (SP)							
**Semester of Enrollment**							
2nd semester, 2020	1,364	45.66	48.98	42.41-55.55	1		
1st semester, 2021	1,614	54.05	24.32	18.03-30.60	0.66	0.51-0.86	< 0.01
2nd semester, 2021	8	0.28	54.43	38.87-69.99	1.99	1.15-3.46	< 0.01

^a^ Adjusted for sex, age group, IBGE skin color categories, region, and household size (approx. equal proportion; ~35% ≥6 persons), and semester of enrollment.

**FIGURE 1:**
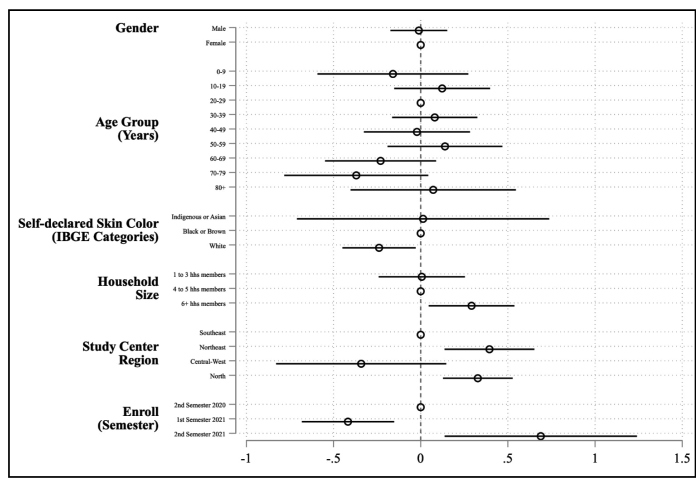
Prevalence ratios (PRs) and 95% confidence intervals (CI) for SARS-CoV-2 from a survey-weighted Poisson regression model considering age, sex, IBGE-defined skin color, household size, and region of residence. **Note:** Each circle represents the estimated PR from the survey-weighted Poisson regression model, and the horizontal lines indicate the corresponding 95% CI. The empty (hollow) circle marks the reference category for each variable. The vertical dashed line represents the null value (PR = 1); CIs that cross this line suggest no evidence of an association (analogous to p ≥ 0.05).

The median age was 43.70 years (IQR 25-60 years). Among the nine age groups, individuals aged 20-29 years were the largest group (15.73%) and those aged ≥ 80 years (4.65%) were the smallest. Most participants were female (58.93%). No significant differences in seroprevalence were observed between females and males or between age groups. The self-reported skin color categories followed the Brazilian Institute of Geography and Statistics classification^15^. Most participants self-identified as Black or Brown (73.17%). Participants who self-identified as White had a 22% lower prevalence than those who identified as Black or Brown (PR = 0.78, 95% CI 0.63-0.97; Figure 1 and Table 1). The average household comprised five individuals. The household size of the respondents was used to divide the participants into three groups of approximately the same proportion: a) households with three or fewer members, b) households with between four and five members, and c) households with six or more members. About 35% of households had six or more people living in them. Relative to households with four to five members, those with six or more family members had a higher PR (PR= 1.34, 95% CI 1.05-1.71). 

**FIGURE 2:**
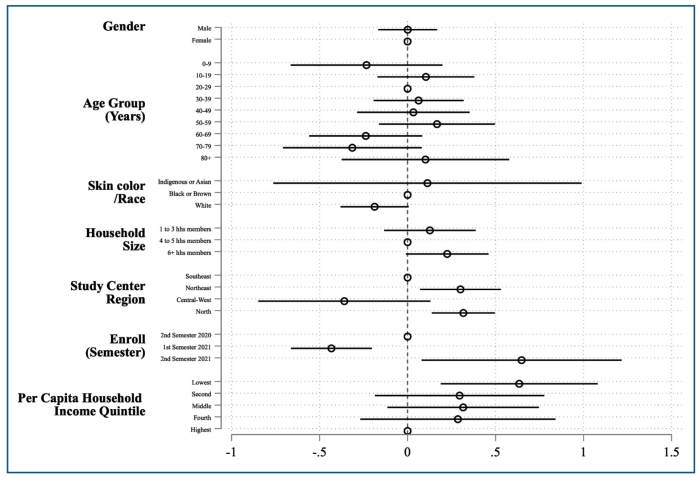
Prevalence ratios (PRs) and 95% confidence intervals (CI) for SARS-CoV-2 from a survey-weighted Poisson regression model considering per capita household income. **Note:** Each circle represents the estimated PR from the survey-weighted Poisson regression model, and the horizontal lines indicate the corresponding 95% CI. The empty (hollow) circle marks the reference category for each variable. The vertical dashed line represents the null value (PR = 1); CIs that cross this line suggest no evidence of an association (analogous to p ≥ 0.05).

Seroprevalence among households, stratified by per capita household income quintiles, controlling for the individual’s age group, semester of enrollment, region, self-identified sex, and IBGE skin color categories, is reported in Table 2. Household income data were calculated for the nine study centers, except for São Jose Ribeirao Preto, which did not collect household income data for the participants. The individual mean per capita income was R$646 (approximately US$121) based on self-reported household income. After adjusting for sex, age, IBGE-defined skin color categories, household size, region, and enrollment semester, individuals in the lowest per capita income quintile had an 88% higher seroprevalence (PR = 1.89, 95% CI 1.21-2.95) compared with individuals in the highest income quintile (Figure 2).

**TABLE 2:** Prevalence and prevalence ratios of SARS-CoV-2 and income vulnerability, AVISA.

Characteristic	Participants		Prevalence		Prevalence Ratio^a^		P-value^a^
	n	Weighted (%)	Weighted (%)	95% CI	Weighted (%)	95% CI	
Household Income per capita Quintile n= 2,666^b^							
Lowest	562	21.06	53.58	44.97-62.18	1.89	1.21-2.95	0.01
Second	549	20.58	38.55	28.23-48.87	1.35	0.83-2.17	0.23
Third	528	19.82	33.18	25.81-40.54	1.37	0.89-2.11	0.15
Fourth	510	19.13	32.85	21.99-43.70	1.33	0.77-2.32	0.31
Highest	518	19.41	22.35	12.29-32.41	1		

^a^ Adjusted for sex, age group (nine categories), IBGE skin color categories, region (four regions), and household size (approx. equal proportion; ~35% ≥6 persons), and semester of enrollment (divided into three groups). ^b^ Income was not collected for participants from the Rio Preto study center, and for one participant in Brasilia. Therefore, these participants were excluded from Table 2.

**FIGURE 3:**
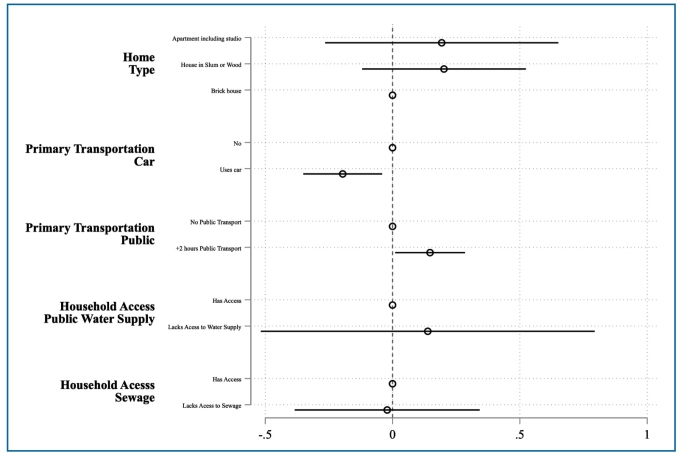
Prevalence ratios (PRs) and 95% confidence intervals (CI) for SARS-CoV-2 from a survey-weighted Poisson regression model considering residence construction type, transportation mode and access to water and sanitation services. **Note:** Each circle represents the estimated PR from the survey-weighted Poisson regression model, and the horizontal lines indicate the corresponding 95% CI. The empty (hollow) circle marks the reference category for each variable. The vertical dashed line represents the null value (PR = 1); CIs that cross this line suggest no evidence of an association (analogous to p ≥ 0.05).

We also analyzed seroprevalence among households by considering structural and infrastructural vulnerabilities. Specifically, we measured the correlation between material living conditions and access to essential services, which strongly affect a household’s resilience to social and economic shocks. Table 3 reports the PRs considering residence type (slum or wood-dwelling, brick home, and/or apartment), reliance on public transportation, use of a car, and whether the residence had access to a public water supply and sewage system, while controlling for the individual’s age group, semester of enrollment, region, sex, and IBGE-defined skin color categories. Individuals who reported using public transportation for > 2 h per day were more likely to test positive for SARS-CoV-2 antibodies (PR=1.17, 95% CI 1.02-1.34). In contrast, holding all else constant, those who used a private automobile had a lower presence of SARS-CoV-2 antibodies (PR=0.82, 95% CI 0.71-0.96) (Figure 3).

**TABLE 3: Prevalence:** and prevalence ratios of SARS-CoV-2 and material vulnerability and access to essential services, AVISA.** **

Characteristic	Participants		Prevalence		Prevalence Ratio^a^		P-value^a^
	n	Weighted (%)	Weighted (%)	95% CI	Weighted (%)	95% CI	
**Type of Home**							
Slum or Wood House	375	7.11	38.37	29.36-47.38	1.26	0.91-1.74	0.16
Brick House	2,399	80.35	35.13	29.22-41.04	1		
Apartment	212	12.54	36.86	19.02-54.70	1.22	0.79-1.90	0.38
**Access to Public Water Supply**							
No access	118	3.97	48.61	29.96-40.29	1.14	0.59-2.19	0.70
Has access	2,868	96.03	35.13	27.01-70.21	1		
**Access to Sewage**							
No access	215	7.21	47.89	37.04-58.74	0.97	0.68-1.40	0.89
Has access	2,771	92.79	34.72	29.22-40.20	1		
**Use Transportation**							
Public Transportation							
Use (at least 2 hours per day)	1,330	44.54	41.60	36.02-47.19	1.17	1.02-1.34	0.03
Doesn’t use or uses less than 1 hour	1,656	55.46	30.89	25.76-36.03	1		
Car							
Use	1,597	53.49	29.81	23.45-36.17	0.82	0.71-0.96	0.01
Doesn’t use	1,389	46.51	42.39	37.56-47.23	1		

Note: ^a^ Adjusted for sex, age group (nine categories), IBGE skin color categories, region (four regions), household size (approx. equal proportion; ~35% ≥6 persons), and semester of enrollment (divided into three groups).

Among participants with detectable SARS-CoV-2 infection, 46.74% had at least one comorbidity, and 26.90% were diagnosed with hypertension (Supplementary Material 1). Approximately one-quarter of the participants were considered obese based on the body mass index criteria calculated from self-reported weight and height (26.41%), although this information was unavailable for 2.88% of the participants (n=86). Moreover, 34.52% of participants reported continuous medication use. Less than 2% of the respondents reported having cancer, and these individuals had a lower prevalence (PR= 0.49, 95% CI 0.25-0.95). Among the patients reporting cardiovascular disease (5%), a higher prevalence of infection was observed (PR=1.37, 95% CI 1.07-1.75) (Table S4, Supplementary Materials). 

To assess sensitivity, we analyzed the seroprevalence, excluding individuals without RBD or IGT results (n=247). In those individuals with RBD and IGT results (n=2,793), the weighted seroprevalence was estimated at 33.22% (95% CI: 31.06-41.38) (Supplementary Material 1). Supplementary Material 1 presents the PRs and 95% CI for SARS-CoV-2 from a survey-weighted Poisson regression model, controlling for age, sex, IBGE-defined skin color, household size, and region of residence, among individuals with ECLIA results in the AVISA Study. No statistically significant differences were observed compared to the full sample of participants.

## DISCUSSION

When the final participants were enrolled in AVISA in October 2021, Brazil was one of the leading countries for SARS-CoV-2 infections and had already registered almost 600 thousand deaths. The magnitude of the COVID-19 pandemic toll in Brazil was most likely underestimated because the testing capacity in Brazil was limited throughout the period analyzed in this study^16^. Even in the wealthiest state in the Brazilian Federation, PCR-confirmed cases represent a minor proportion of confirmed infections, especially in more vulnerable populations. Thus, the actual COVID-19 burden remained underestimated throughout this period. Seroprevalence studies, such as the present study, provide more accurate estimates of infection rates to inform public health responses.

In the context of extreme inequality, reliance on informal employment, poverty, and the absence of vaccination, Brazilian households struggled to withstand the severe decline in economic activity and upsurge in unemployment resulting from the outbreak of COVID-19 cases and deaths throughout 2020 and 2021^17^. Recognizing the severe hardships faced by Brazilian households and firms, national, state, and local governments have introduced programs to aid affected households. Among these programs, the Emergency Aid Program (*Auxilio Emergencial)* was launched by the federal government to provide household cash transfers. In 2020, 67 million households received emergency assistance, which helped supplement labor and pension incomes. In 2021, the program targeted 39.4 million individuals. Despite these aid programs, most individuals who worked in the service sector and were informally employed had limited opportunities to engage in remote work^18^. Even after vaccination against SARS-CoV-2 was introduced in January 2021, vaccines were available for a limited part of the population. As a result, the rollout of vaccination slowly progressed over 2021 until sufficient supplies were procured by the federal government. 

In this study, a high prevalence of infection (35.66%) was observed at baseline among the study population who were enrolled in the primary public health system and reported an income well below the mean per capita in the municipalities participating in the study Supplementary Material 1). In Brazil, official estimates suggest that approximately 65% of households are covered by the SUS primary healthcare model^12^. Among the municipalities participating in the study, the percentage of the municipal population covered by the ESF was highest in Laranjeiras (100%), followed by Belo Horizonte (80.5%), Fortaleza (55.8%), and Porto Velho (51.5%). The national income per capita was R$1,380 (approximately US$258) in 2020. Among the households participating in the study, the average per capita household income (R$577) was below the national average Supplementary Material 1). 

Social inequalities in Brazil, exemplified by precarious housing conditions and a lack of basic sanitation, have contributed to higher COVID-19 mortality rates in socioeconomically disadvantaged communities, as observed in a study performed in Aracaju, Northeastern Brazil^19^. Furthermore, in neighborhoods with lower living condition indices, the COVID-19 case fatality rate was twice as high as that in neighborhoods with better living conditions, underscoring the link between social inequality and disease severity. These findings are consistent with our results and reinforce the disproportionate impact of the pandemic on vulnerable populations.

Studies conducted during health emergencies need to consider the specific vulnerabilities faced by populations that access public health hospitals and clinics and guide public health strategies to protect these populations. In Sergipe, Northeast Brazil, a population-based study reported a seroprevalence of 9.3% (95% CI 8.5-10.1) for SARS-CoV-2 antibodies, highlighting disparities in infection rates between metropolitan areas (11.7%) and smaller municipalities (8.0%)^20^. A survey of the prevalence of anti-SARS-CoV-2 antibodies in a relatively small sample of outpatients of a large public university hospital in São Paulo, Brazil, the epicenter of the pandemic, over six weeks from June 30th to August 4th, 2020, identified a seroprevalence of 13.9%^21^. Our study reported PRs that were 2.6 times higher. The relatively higher SARS-CoV-2 infection rates detected in our study help elucidate the overwhelming challenges faced by the the Brazilian public health system throughout the pandemic. These massive pressures led to the collapse of the Brazilian healthcare system in some cities during the second wave^22^.

Urbanization resulting from crowding during mass transit increases the risk of epidemic disease transmission^23^. In population-based studies, the use of public transit is generally associated with higher seropositivity, race, and ethnicity. However, a significant limitation of these findings is that the results could not be generalized owing to several confounding factors. Policymakers and planners in urban centers with busy transit systems should consider public transport as a risk factor when planning, implementing, and monitoring pandemic strategies in the event of future infectious diseases outbreaks^24^ ^,^ ^25^.

A systematic review of serosurveys in Brazil estimated the seroprevalence to be 11.0%. Among the studies identified, 18 population-based surveys were conducted between 2020 and 2021. Based on these studies, the seroprevalence was estimated to be 9% (95% CI 6.0‒9.0)^26^. In comparison to these studies, the increased seropositivity reported in this study can be attributed to some factors. First, the improved test performance and increased accessibility of the two different types of serological tests may have contributed to more accurate case detection and contact tracing. Second, the emergence of novel viral variants is also a significant contributing factor. Five variants of concern (alpha, beta, gamma, delta, and omicron) have emerged and replaced the wild-type variant, thus influencing transmission dynamics. Third, there were significant differences in study design (e.g., follow-up duration, testing, and monitoring protocols) and heterogeneity effects, warranting readers to be cautious when interpreting and comparing the findings of this study with other seroprevalence studies in Brazilian households.

This study had some limitations. The primary driver of municipality selection was that the study center was a pre-qualified Butantan partner. Thus, the generalizability of the results is limited, particularly because no study centers in southern Brazil were included. Owing to the complexity of public health emergencies, the 11 municipalities that participated in the study recruited participants in different months of 2020 and 2021. Recruitment progressed over several months in most municipalities. Each city required an average of 80 days to enroll participants, reaching a target of 320. This temporal heterogeneity may have influenced the results, making it challenging to compare locations and time periods.

The self-reported data collected during the initial intake interview were susceptible to recall and measurement biases. Prior infections and prophylactic medications were self-reported. Both were used as exclusion criteria. Height, weight, comorbidities, household income, number of household members, sex, means of transportation, adherence to masks, social distancing, and skin color were also self-reported. 

Some information was not collected from the entire sample; therefore, it was not used in the study. For example, of the 2,435 adults enrolled in the study, information regarding occupation at the time of recruitment was only provided by 55.7%. In part, the lack of data on employment status may be because many of these individuals have lost their jobs or were employed only informally. Individuals were also not asked to provide information on whether they received supplemental assistance from government programs, such as the Emergency Aid Program.

In this study, we decided to model prevalence at the individual rather than household level. Family members could opt not to participate in the research; as a result, there were household members whose serostatus was unknown. Because the prevalence ratio at the household level does not necessarily reflect the actual prevalence among family members, we did not analyze data based on the number of household members who tested positive. In future studies, we plan to explore the PR within and across households.

Our findings indicate that a relatively high proportion (approximately 36%) of the Brazilian population dependent on the public health system (around 65%) had acquired detectable antibodies to SARS-CoV-2 by 2021 Supplementary Material 1. Furthermore, vulnerable households in Brazil have a higher prevalence of SARS-CoV-2 infection. This evidence is important for informing decision makers about preparedness for epidemics or pandemics similar to COVID-19. Integrating more robust and widespread surveillance systems will help track infection rates in real-time, thereby enabling timely responses to emerging threats. A deeper analysis of the social determinants of health, particularly in vulnerable populations, is essential to understand the full impact of pandemics on different social groups and to design tailored interventions that minimize health inequities.

Furthermore, exploring the use of surveillance tools for better monitoring and early detection of infectious diseases at both individual and community levels is critical. The ability to rapidly scale diagnostic capabilities, such as accessible and affordable testing, will enable faster identification of cases and prevent the widespread transmission of diseases. 

## Data Availability

The data that support the findings of this study are available on request from the corresponding author.
