# Supplementary material for: Prevalence of SARS-CoV-2 Infection Among Vulnerable Populations Relying on Public Health Services: Findings from the AVISA Study in Brazil
Source: Rev Soc Bras Med Trop. 2026 Feb 16;59:e0151-2025. doi: 10.1590/0037-8682-0151-2025 (PMC12904596; doi:10.1590/0037-8682-0151-2025)
Supplement: Supplementary Material 1 [file 1678-9849-rsbmt-59-e0151-2025-md1.pdf]

Supplementary Materials

"Prevalence of SARS-CoV-2 Infection Among Vulnerable Populations Relying on Public Health Services: Findings from the AVISA Study in Brazil"

December 2025

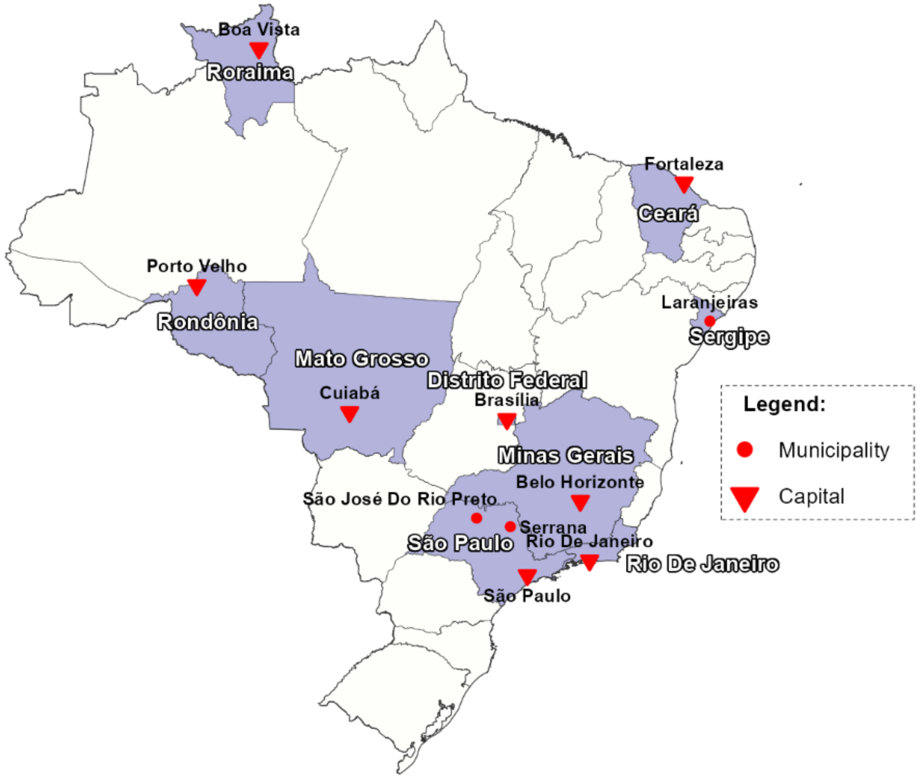

Figure S1. Distribution of the study participating municipalities across four geographical regions in Brazil.

Table S1. Population, FHP Coverage, per Capita Income for each municipality, and Mean Household Income and Baseline Prevalence for SARS-COV-2 in the AVISA cohort

Region and City	Population[1] (2021)	Coverage of FHP[2] (2021) (%)	Per capita Income[3] (2020) Brazilian reais (BRL)	Per capita income of Households in the AVISA Study	Baseline Prevalence for SARS-CoV-2 in the AVISA study (95% CI)
**Central-West**					
Brasília-DF*	3,094,325	43.0	2,930	646	52.0 (35.6-68.4)
Cuiabá-MT*	623,614	41.1	2,241	687	21.9 (12.5-31.3)
**North**					
Boa Vista-RR*	436,591	47.5	1,282	679	55.6 (45.5-65.7)
Porto Velho-RO*	548,952	51.5	1,401	565	40.8 (32.8-48.9)
**Northeast**					
Fortaleza-CE*	2,703,391	55.8	2,102	636	55.6 (45.0 - 66.3)
Laranjeiras-SE	30,327	100.0	1,994**	434	29.9 (14.9-44.9)
**Southeast**					
Belo Horizonte-MG*	2,530,701	80.5	2.533	880	23.8 (16.9-30.7)
Rio de Janeiro-RJ*	6,775,561	44.1	2,929	498	44.2 (38.1-50.3)
São José do Rio Preto-SP	469,173	44.9	3,014**	--***	18.2 (11.2 - 25.2)
Serrana-SP	46,166	30.6	3,014**	789	23.6 (21.2- 26.0)
**National**	213.3 millions	65.1	1,380	1,380	

Notes: *State Capital ** Per Capita Income for State Capital. ***Household Income Data were not collected in this city.

Sources:

1. Instituto Brasileiro de Geografia e Estatística (IBGE). Tabelas de estimativas de população para 1º de julho de 2021 [Internet]. [Accessed 2024 December 14].
2. Instituto Brasileiro de Geografia e Estatística (IBGE). Pesquisa nacional de saúde 2019. Informações sobre domicílios, acesso e utilização dos serviços de saúde: Brasil, grandes regiões e unidades da federação[Internet]. [Accessed 2024 December 14].
3. Instituto Brasileiro de Geografia e Estatística (IBGE). Rendimento domiciliar per capita médio (SIS/IBGE).

Table S2. Enrollment by Month

Month	n	Percent
October 2020	136	4.6
November 2020	980	32.8
December 2020	942	31.6
January 2021	476	16.0
February 2021	277	9.2
March 2021	99	3.3
June 2021	8	0.3
July 2021	35	1.2
August 2021	20	0.7
September 2021	9	0.3
October 2021	4	0.1
Total	2,986	100

Table S3. Enrollment by Study Center

Center	n	Percent
Belo Horizonte	333	11.2
Boa Visita	317	10.6
Brasilia	76	2.6
Cuiaba	319	10.7
Fortaleza	338	11.3
Laranjeiras	338	11.3
Porto Velho	311	10.4
Rio de Janeiro	317	10.6
Sao Jose de Rio Preto	319	10.7
Serrana	320	10.7
Total	2,986	100

Table S4. Prevalence and prevalence ratios of SARS-CoV-2 and self-reported clinical diseases

Comorbidities	Participants		Prevalence		Prevalence Ratio[a]		P-value[a]
	n	Weighted %	Weighted (%)	95% CI	Weighted (%)	95% CI	
Smoker	213	0.1	27.7	18.6 - 36.8	0.7	0.5 - 1.0	0.06
Diabetes mellitus	66	0.1	33.4	24.1 - 42.7	1.0	0.7 - 1.3	0.72
Cardiovascular disease	20	0.1	36.3	23.3 - 49.3	1.3	1.0 - 1.7	0.03
Hypertension	374	0.3	30.8	24.3 - 37.2	1.1	0.9 - 1.4	0.35
Asthma	101	0.1	36.6	26.1 - 47.0	1.3	0.9 - 1.8	0.11
Chronic neurological disease	49	0.0	36.9	20.2 - 53.6	1.2	0.8 - 1.8	0.44
Cancer	21	0.0	23.9	6.9 - 40.9	0.5	0.3 - 1.0	0.04
Chronic liver disease	65	0.0	43.9	22.0 - 65.8	1.0	0.6 - 1.8	0.99
Immunodepression /Immunodeficiency	36	0.0	34.2	9.6 - 58.9	1.0	0.5 - 2.1	0.93
Chronic kidney disease	101	0.0	28.5	3.1 - 53.8	1.0	0.5 - 1.8	0.94
Chronic obstructive pulmonary disease	33	0.0	39.7	13.0 - 66.3	1.4	0.8 - 2.4	0.21
Chronic hematologic disease	18	0.0	32.6	4.1- 61.2	1.0	0.5 - 2.0	0.98
Other comorbidities	107	0.0	33.5	18.1 - 48.9	1.0	0.7 - 1.5	0.94
Obesity (BMI* ≥ 30 kg/m²) (N = 2,884)	766	0.3	34.8	29.3 - 40.3	1.0	0.8 - 1.2	1.00
Medication of continuous use	1,030	0.4	29.5	23.6 - 35.5	0.8	0.6 - 1.0	0.09

Notes:
[a] Adjusted for sex, age group, skin color, region, household size and semester of enrollment.
* BMI: Body Mass Index. Individuals with BMI < 12 and BMI > 300 and pregnant women were excluded.

Table S5. Prevalence and prevalence ratios of SARS-CoV-2 by sociodemographic characteristics for (1) Rapid and/or ECLIA results and (2) ECLIA-only results, AVISA

Characteristic	Individuals with ECLIA and/or RAPID TEST				Only Individuals with ECLIA results			
	Participants		Prevalence		Participants		Prevalence	
	n	Weighted (%)	Weighted (%)	95% CI	n	Weighted (%)	Weighted (%)	95% CI
**Overall**	2,986	100	35.66	30.55 – 40.77	2,739	100	33.22	31.06 – 41.38
**Sex**								
Male	1,226	41.07	34.46	28.50 – 40.41	1,130	41.26	34.81	28.75 – 40.87
Female	1,760	58.93	36.50	30.81 – 42.20	1,609	58.74	37.20	31.42 – 42.99
**Age Group (years)**								
0–9	167	5.61	30.97	18.04 – 43.89	156	5.70	31.15	18.13 – 44.17
10–19	330	11.05	40.42	31.95 – 48.89	300	10.96	41.49	32.73 – 50.25
20–29	470	15.73	36.63	27.97 – 45.30	435	15.88	37.11	28.39 – 45.82
30–39	382	12.83	41.23	33.19 – 49.29	346	12.65	42.64	34.72 – 50.55
40–49	438	14.67	35.30	25.30 – 45.32	402	14.68	35.55	25.37 – 45.74
50–59	464	15.53	40.65	31.53 – 49.77	424	15.46	41.70	32.50 – 50.91
60–69	404	13.52	28.02	21.27 – 34.77	375	13.67	28.16	21.33 – 34.99
70–79	192	6.41	23.03	14.14 – 31.93	174	6.36	23.56	14.41 – 32.71
80+	139	4.65	35.45	19.68 – 51.21	127	4.64	35.34	18.51 – 50.16
**Self-reported skin color/race using IBGE categories**								
Brown and Black	2,185	73.17	38.30	32.85 – 43.75	1,996	72.88	39.76	33.48 – 44.48
White	762	25.53	27.90	21.31 – 34.49	707	25.80	28.23	21.57 – 34.89

Asian and Indigenous	39	1.29	39.76	2.56 – 76.96	36	1.32	39.76	2.55 – 76.97
**Size of Household**								
1-3 members	950	31.81	31.44	24.54 – 38.33	869	31.74	31.86	24.85 – 38.87
4-5 members	997	33.39	32.52	24.88 – 40.17	909	33.19	33.12	25.39 – 40.86
6+ members	1,039	34.80	42.54	34.54 – 50.55	961	35.06	43.09	34.96 – 51.21
**Region**								
Central-West<br>Cuiabá (MT) and Brasília (DF)	94	3.15	24.75	14.55 – 34.94	66	2.41	30.27	19.13 – 41.41
North<br>Boa Vista (RR) and Porto Velho (RO)	234	7.84	50.17	43.51 – 56.82	214	7.82	51.10	44.44 – 57.77
Northeast<br>Fortaleza (CE) and Laranjeiras (SE)	738	24.70	55.52	44.91 – 66.14	681	24.85	55.83	45.00 – 66.66
Southeast<br>Belo Horizonte (MG)<br>Rio de Janeiro (RJ)<br>São José do Rio Preto (SP) and Serrana (SP)	1,920	64.31	26.80	21.26 – 32.34	1,778	64.92	27.13	21.53 – 32.74
Semester of Enrollment								

2nd semester, 2020	1,364	45.66	48.98	42.41 – 55.55	1,238	50.19	50.12	43.45 – 56.80
1st semester, 2021	1,614	54.05	24.32	18.03 – 30.60	1,493	54.52	24.59	18.23 – 30.96
2nd semester, 2021	8	0.28	54.43	38.87 – 69.99	8	0.29	54.56	38.94 – 70.17

Figure S2. Prevalence ratios (PRs) and 95% confidence intervals for SARS-CoV-2 from a survey-weighted Poisson regression model considering age, gender, IBGE-defined skin color, household size, and region of residence only for individuals with ECLIA results in the AVISA Study

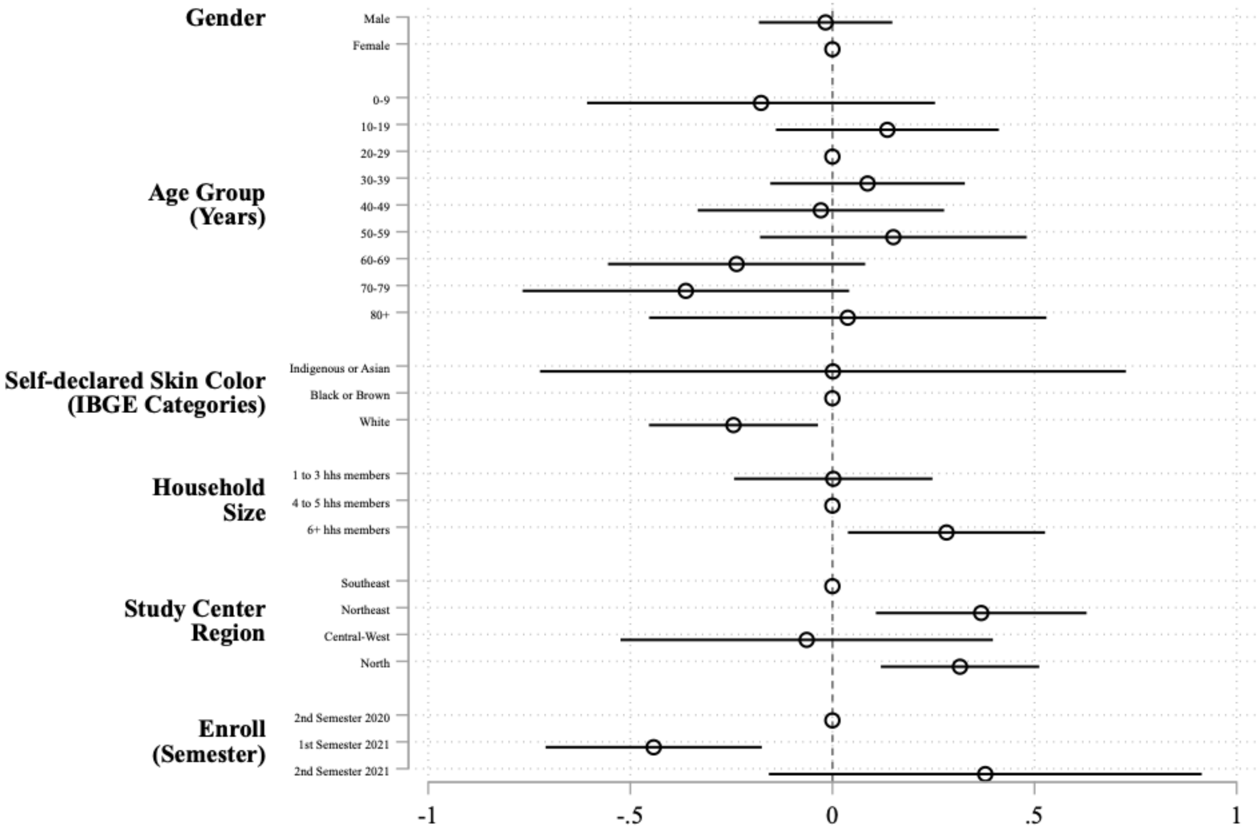

Note: Each circle represents the estimated prevalence ratio (PR) from the survey-weighted Poisson regression model, and the horizontal lines indicate the corresponding 95% confidence intervals. The empty (hollow) circle marks the reference category for each variable. The vertical dashed line represents the null value (PR = 1); confidence intervals that cross this line suggest no evidence of an association (analogous to $p \geq 0.05$).

Table S6. Questionnaire Administered during Initial Visit

Data entrevista: d d m m a a   Id Domicilio: Id Participante:

COV-01-IB
Versão 1.1 de 04-Set-2020
Página 1

Estudo AVISA
Avaliação geral
CONFIDENCIAL

INSTITUTO BUTANTAN
A serviço da vida

Avaliação de incidência de infecção por SARS-CoV-2 e de COVID-19 no Brasil – COV- 01-IB
Estudo AVISA

FORMULÁRIO – MÓDULO DO PARTICIPANTE: NA INCLUSÃO OU ATUALIZAÇÃO
Inicial: Atualização:

I. IDENTIFICAÇÃO E CONTATO DO PARTICIPANTE

A informação desta seção é Confidencial e, portanto, não será registrada na base de dados

Nome do participante: ____________ CPF
Nome da mãe do participante: ____________
Telefone celular 1   Tem WhatsApp?   Telefone celular 2   Tem WhatsApp?
E-mail: ____________
No. do SIVEP_Gripe (se houver): ____________

II. DEMOGRÁFICOS

1. Data de nascimento d d m m a a   2. Sexo: Masculino ☐ Feminino ☐ Ignorado ☐
3. Como se classifica em termos de raça? Branca ☐ Parda ☐ Preta ☐ Amarela ☐ Indígena ☐
4. É gestante? Sim ☐ Não ☐ Ignorado ☐ Na ☐   4.a. Se sim, quantas semanas de gestação? ______
5. O participante:
   - Estuda ○
   - Trabalha ○   Qual é ocupação principal do participante?
   - No aplica ☐   Código CBO da ocupação ☐   Código CNAE de atividade económica: Seção: ☐ Divisão: ☐
6. Quantas pessoas dormem no mesmo cômodo do participante? ☐

III. ANTECEDENTES DO PARTICIPANTE

	Não	Sim	Ig/NA		Não	Sim	Ig/NA
7. É fumante?	N	S	Ig	14. Tem doença neurológica crônica?	N	S	Ig
8. Tem doença cardiovascular?	N	S	Ig	15. Tem doença pulmonar obstrutiva crônica?	N	S	Ig
9. Tem hipertensão arterial sistêmica?	N	S	Ig	16. Tem doença renal crônica?	N	S	Ig
10. Tem doença hematológica crônica?	N	S	Ig	17. Tem imunodepressão/imunodeficiência?	N	S	Ig
11. Tem doença hepática crônica?	N	S	Ig	18.Tem câncer?	N	S	Ig
12. Tem asma?	N	S	Ig	19. Tem outro fator de risco/comorbidade?	N	S	Ig
13. Tem diabetes mellitus?	N	S	Ig	20. Faz uso de alguma medicação de uso contínuo?	N	S	Ig

Em caso afirmativo em alguma das questões 16 a 19, por favor, especificar CID-10 do diagnóstico principal   CID 10

16. ____________
17. ____________
18. ____________
19. ____________

Ig/NA ignorado ou não aplica

Observações:

Nome e rubrica do avaliador: ____________

Data entrevista: d d m m a a Id Domicilio: Id Participante:

COV-01-IB
Versão 1.1 de 04-Set-2020
Página 1

Estudo AVISA
Avaliação de rotina
CONFIDENCIAL

INSTITUTO BUTANTAN
A serviço da vida

FORMULÁRIO – MÓDULO DO PARTICIPANTE: AVALIAÇÃO DE ROTINA

Deve ser realizado na inclusão do participante e em todas as visitas

I. DADOS DE CONTIGENCIA NAS ÚLTIMAS QUATRO SEMANAS

Para as perguntas seguintes, considere a definição:
CONTATO: Estar num ambiente fechado com uma pessoa por mais de 15 minutos ou estar conversando ou interagindo a menos de um metro de distância.

1. Sou um paciente COVID-19 confirmado por um teste de laboratório: Sim ☐ Não ☐ Ignorado ☐
2. Na última semana, em média, quantas vezes você saiu de casa: ☐
3. Sobre a quarentena:
   - Minha família e eu estamos todos em quarentena, nos mesmos quartos ☐
   - Estou em quarentena da minha família, em uma sala separada ☐
   - Não estou em quarentena ☐
4. Como tem sido a rotina de atividades durante o distanciamento social, fora de atividades de trabalho ou estudo?
   - Fica em casa o tempo todo ☐
   - Sai apenas para atividades essenciais (ex. comprar comida) ☐
   - Sai de vez em quando para alguma atividade ☐
   - Sai todos os dias, para atividades regulares ☐
5. Quantos dias da semana, em média, você:
   - Ficou em casa o dia inteiro ☐
   - Saiu para fazer compras no mercado/supermercado ☐
   - Saiu de casa para ir a um serviço médico/consulta médica ☐
   - Saiu para fazer atividade física ou se movimentar ☐
   - Saiu de casa para visitar familiares e/ ou amigos ☐
   - Saiu de casa para ir a bancos ou lotéricas ☐
   - Saiu de casa para algum culto/missa/evento religioso ☐
   - Saiu de casa para as atividades educacionais ☐
   - Saiu de casa para trabalhar ☐
6. Pensando na rotina da casa durante o período de distanciamento social, quem tem entrado na casa?
   - Só os familiares que moram junto, se tiver, e mais ninguém ☐
   - Alguns parentes próximos visitam 1 a 2 vezes por semana ☐
   - Alguns parentes próximos visitam quase que todos os dias ☐
   - Amigos, parentes ou outros que visitam 1 a 2 vezes por semana ☐
   - Amigos, parentes ou outros que visitam quase todos os dias ☐
7. Ao sair de casa você teve contato com pessoas (que não moram em sua casa)
   Selecione uma opção de A a F ☐
8. Ao sair de casa você utilizou máscara?
   Selecione uma opção de A a F ☐
9. Utilizou o transporte público coletivo para se deslocar? (ônibus, metrô ou similar)
   Selecione uma opção de A a F ☐

A Nunca
B Muito pouco (até1 hora)
C Pouco (2-3 horas)
D Mais ou menos (4-5 horas)
E Bastante (mais de 6 horas)
F Sempre

10. Meio que utiliza para se deslocar
    Metrô ○ Ônibus ○ Bicicleta/Moto ○ A pé ○ Carro próprio ○ Barco ○
11. Você viajou (saiu da sua cidade para ir à outra)?
    Lazer ○ Urgência ○ Trabalho/Estudo ○ Não viajei ○

Nome e rubrica do avaliador: ____________________

Data entrevista: | d | d | m | m | a | a | Id Domicílio: | | | | Id Participante: | | | | |

ANEXO A: ATENDIMENTO CLÍNICO AMBULATORIAL

1. Em que tipo de serviço de saúde?
- Posto ou unidade básica de saúde
- Serviço ambulatorial generalista
- Serviço ambulatorial especializado
- Pronto Atendimento ou Pronto Socorro
- Hospital ou Clínica
- Outro serviço
- Especifique:

2. Data do atendimento ambulatorial:
Início: d d m m a a
Termino: d d m m a a

3. O atendimento foi por síndrome gripal?
Sim   Não   Ignorado

4. Teve queda da saturação de oxigênio menor que 95,0%?
Sim   Não   Ignorado

5. O participante fez tratamento específico contra SARS-CoV-2/COVID-19?
Sim   Não   Ignorado   Especifique:

6. Coletou amostra de secreção respiratória para PCR-RT?
Sim   Não   Ignorado

7. Caso afirmativo, data da coleta da secreção respiratória
d d m m a a

8. Caso afirmativo, qual foi o resultado?
- SARS-CoV-2 ○   Influenza ○   Vírus Sincicial Respiratório ○
- Outro vírus respiratório ○   Outro agente ○   Ignorado ☐
- Especifique: ____________________

9. Classificação final do diagnóstico de atendimento ambulatorial:
- COVID-19
- Influenza
- Síndrome Gripal por vírus sincicial respiratório ou bronquiolite
- Síndrome Gripal por outro vírus respiratório
- Outro diagnóstico
- Especifique: ____________________

CID-10 do diagnóstico principal: [ ]

Observações:

Nome e rubrica do avaliador: ____________________

Data entrevista: d d m m a a   Id Domicílio: ☐☐☐   Id Participante: ☐☐☐☐

ANEXO B: ATENDIMENTO CLÍNICO HOSPITALAR

1. Local de internação hospitalar? *(Nome do hospital)* ____________________

CNES: ☐
Município: ____________ UF: ☐

2. Datas de internação:
Início: d d m m a a
Término: d d m m a a

3. A internação hospitalar foi por Síndrome Respiratória Aguda Grave? Sim ☐ Não ☐ Ignorado ☐

4. O participante fez tratamento específico contra SARS-CoV-2/COVID-19? Sim ☐ Não ☐ Ignorado ☐
Especifique: ____________________

5. O participante foi internado na UTI
Sim ☐ Não ☐ Ignorado ☐

6. Datas de Internação na UTI:
Início: d d m m a a
Término: d d m m a a

7. Uso de suporte ventilatório: Sim, invasivo ☐ Sim, não invasivo ☐ Não ☐ Ignorado ☐

8. Coletou amostra de secreção respiratória para PCR-RT?
Sim ☐ Não ☐ Ignorado ☐

9. Caso afirmativo, data da coleta da secreção respiratória
d d m m a a

10. Caso afirmativo, qual foi o resultado?
SARS-CoV-2 ☐ Influenza ☐ Vírus Sincicial Respiratório ☐
Outro vírus respiratório ☐ Outro agente ☐ Ignorado ☐
Especifique: ____________________

11. Evolução do participante
Recuperado ☐ Recuperado com sequelas ☐
Óbito ☐ Ignorado ☐

12. Data de evolução do participante
d d m m a a

13. Classificação final do diagnóstico de internação hospitalar:
Síndrome Respiratória Aguda Grave por SARS CoV-2/COVID-19 ☐
Síndrome Respiratória Aguda Grave pelo vírus Influenza ☐
Síndrome Respiratória Aguda Grave por vírus sincicial respiratório ☐
Síndrome Respiratória Aguda Grave por outro vírus respiratório ☐
Síndrome Respiratória Aguda Grave de outra etiologia ☐
Síndrome Respiratória Aguda Grave etiologia ignorada ☐
Outro diagnóstico ☐
Especifique: ____________________

CID-10 do diagnóstico principal: ☐

Observações:

Nome e rubrica do avaliador: ____________________

Data entrevista: | d | d | m | m | a | a | Id Domicílio: [ ][ ][ ]

COV-01-IB
Versão 1.0 de 23-Jun-2020
Página 1

Estudo AVISA
Avaliação geral
CONFIDENCIAL

INSTITUTO BUTANTAN
A serviço da vida

Avaliação de incidência de infecção por SARS-CoV-2 e de COVID-19 no Brasil – COV- 01-IB
Estudo AVISA

FORMULÁRIO - MÓDULO DO GRUPO FAMILIAR
este questionário deverá ser respondido para todo domicílio na primeira visita e quando houver atualizações

Endereço e telefone é informação confidencial e, portanto, não será registrada na base de dados

Inicial: [ ]
Atualização: [ ]

Município: ______________ Estado: [ ] CEP: ______________
Endereço da residência: ______________
Telefono fixo (se houver): ______________

I. DOMICÍLIO

1. Tipo de domicílio:
- Apartamento [ ]
- Apartamento quitinete [ ]
- Casa de alvenaria [ ]
- Casa de madeira [ ]
- Barraco em favela [ ]
- Casa de alvenaria em favela [ ]
- Casa de cômodo (pensão ou cortiço) [ ]
- Outros, especifique [ ]

____

2. Utiliza água de abastecimento público? Sim [ ] Não [ ] Ignorado [ ]
Especifique a fonte de água, se não for do sistema público:
____

3. Está ligada à rede de esgoto? Sim [ ] Não [ ] Ignorado [ ]
Especifique a rede de esgoto, se não for do sistema público:
____

4. Renda familiar (valor em reais): ______________ reais

II. RESIDENTES

5. Qual é o número total de pessoas residentes? [ ]

6. Indique quantos residentes pertencem aos seguintes grupos de idade:
- 0-9 anos [ ]
- 10-19 anos [ ]
- 20-29 anos [ ]
- 30-39 anos [ ]
- 40-49 anos [ ]
- 50-59 anos [ ]
- 60-69 anos [ ]
- 70-79 anos [ ]
- 80 anos ou mais [ ]

7. Quantas pessoa com fator de risco/comorbidade no domicílio [ ][ ]
(Idade > 60 anos, cardiopatia, diabetes mellitus, neuropatia, pneumopatia, imunossupressão, doença hematológica, obesidade, doença hepática, câncer, doença renal)

8. Há pessoas que fumam na residência? Sim [ ] Não [ ] Ignorado [ ]

9. Desde janeiro/2020, quantas pessoas na sua família foram casos suspeitos ou confirmados de COVID-19?
Casos suspeitos [ ] Casos confirmados [ ] Ignorado [ ]

Rubrica do avaliador: ______________
