# Supplementary material for: Prevalence of SARS-CoV-2 Infection Among Vulnerable Populations Relying on Public Health Services: Findings from the AVISA Study in Brazil
Source: Rev Soc Bras Med Trop. 2026 Feb 16;59:e0151-2025. doi: 10.1590/0037-8682-0151-2025 (PMC12904596; doi:10.1590/0037-8682-0151-2025)
Supplement: Supplementary Material 2 [file 1678-9849-rsbmt-59-e0151-2025-md2.pdf]

Supplementary Material 2: AVISA STUDY GROUP

Sponsor	Butantan Institute<br>Av. Vital Brasil, 1500<br>São Paulo – SP<br>Brazil – 05503 900<br>Telephone: 11 2627 9372<br>Fax: 11 2627 9375
Sponsor Representative:	Eliana Nogueira Castro de Barros<br>Manager of Epidemiology<br>Center for Clinical Trials and<br>Pharmacovigilance of Butantan Institute<br>Av. Vital Brasil, 1500<br>São Paulo – SP<br>Brazil – 05503 900<br>Telephone: 11 3723 7024<br>eliana.barros@fundacaobutantan.org.br
Operations Management:	Daniela Haydee Ramos Silveira<br>Center for Clinical Trials and<br>Pharmacovigilance of Butantan Institute<br>Av. Vital Brasil, 1500<br>São Paulo - SP<br>Brazil – 05503 900<br>Telephone: 11 3723 2136<br>daniela.silveira@fundacaobutantan.org.br
Research Coordinator<br><br><br><br><br>Principal Investigator	Dr. Carolina Luisa Alves Barbieri<br>Center for Clinical Trials and<br>Pharmacovigilance of Butantan Institute<br>Av. Vital Brasil, 1500<br>São Paulo - SP<br>Brazil – 05503 900<br>Telephone: 11 3723 6702<br>carolina.barbieri@fundacaobutantan.org.br<br><br>Eliana Nogueira Castro de Barros<br>Manager of Epidemiology<br>Center for Clinical Trials and<br>Pharmacovigilance of Butantan Institute<br>Av. Vital Brasil, 1500<br>São Paulo – SP<br>Brazil – 05503 900<br>Telephone: 11 3723 7024<br>eliana.barros@fundacaobutantan.org.br
Research Center SAO05<br>Faculdade Saúde Santa Marcelina	Principal Investigator<br>Dr. Samuel Soares Filho

Rua Cachoeira Utupanema, 40 – Vila Carmosina<br>ZIP: 08270-140 - São Paulo – SP<br>Telephone: (11)2217-9110	Professor at Faculdade Santa Marcelina and Medical Supervisor of APS Santa Marcelina<br>Rua Cachoeira Utupanema,40 – Vila Carmosina<br>ZIP: 08270-140 - São Paulo – SP<br>Telephone: (11) 95835-1538<br>samuel.soares@santamarcelina.edu.br
Research Center SJP01<br>Faculdade de Medicina de Rio Preto (FAMERP)<br>Av. Brigadeiro Faria Lima, 5416 - Vila São Pedro<br>ZIP 15090-000 - São José do Rio Preto/SP<br>Telephone: 17 3201-5888<br>E-mail: faepe@faepefamerp.org.br	Principal Investigator<br>Dr. Mauricio Nogueira<br>Faculdade de Medicina de São José do Rio Preto, Department of Infectious and Parasitic Diseases<br>Virology Research Laboratory<br>Av Brigadeiro Faria Lima, 5416<br>Green Valley Edge City<br>15090000 - São José do Rio Preto, SP - Brazil<br>Telephone: 17 3201 5731<br>mnogueira@famerp.br
Research Center RAO02<br>Hospital das Clínicas<br>Faculdade de Medicina de Ribeirão Preto (FMRP-USP)<br>Campus Universitário, s/n, -Bairro Monte Alegre,<br>ZIP:14048-90 Ribeirão Preto/SP<br>Telephone: (16) 3963-6492<br>E-mail: upc-hc@hcrp.usp.br	Principal Investigator<br>Dr. Marcos de Carvalho Borges<br>Faculdade de Medicina de Ribeirão Preto-FMRP-USP<br>Division of Clinical Emergencies<br>Department of Internal Medicine<br>Rua Bernardino de Campos 1000- Centro<br>ZIP: 14015-130, Ribeirão Preto<br>Telephone: 16 3987-8800<br>marcosborges@fmrp.usp.br
Research Center BHZ01<br>Hospital das Clínicas<br>UFMG- Belo Horizonte<br>Av. Prof. Alfredo Balena nº 110, Santa Efigênia, Belo Horizonte, ZIP: 30.130.100	Principal Investigator<br>Dr. Mauro Martins Teixeira<br>Federal University of Minas Gerais, Institute of Biological Sciences, Department of Biochemistry and Immunology.<br>Immunopharmacology Laboratory<br>Avenida Antonio Carlos 6627 - Pampulha<br>ZIP: 31270-901 - Belo Horizonte, MG<br>Telephone: 31 3409 2651<br>Fax: 31 3409 2651<br>mmtex.ufmg@gmail.com / mmtex@icb.ufmg.br
Research Center AJU01<br>Federal University of Sergipe<br>Avenida Marechal Rondon, S/n - Jardim Rosa Elze, São Cristóvão - SE, 49100-000<br>ppgcs.ufs@gmail.com	Principal Investigator<br>Dr. Ricardo Queiroz Gurgel<br>Associate Professor of Pediatrics<br>Federal University of Sergipe<br>Avenida Marechal Rondon, S/n - Jardim Rosa Elze<br>São Cristóvão - SE, 49100-000

	Telephone: 79 2105 1787<br>ricardoqgurgel@gmail.com
Research Center FOR01<br>Federal University of Ceará<br>Faculty of Medicine<br>Center for Tropical Medicine Prof. Dr. Joaquim E. Alencar<br>Rua Alexandre Baraúna, 949 - Rodolfo Teófilo<br>ZIP: 60430-160 Fortaleza - Ceará<br>Telephone: 85 3366 8001 / 85 3366 8003	Principal Investigator<br>Dr. Ivo Castelo Branco Coelho<br>Coordinator<br>Center for Tropical Medicine UFC<br>Rua Alexandre Baraúna, 949 - Rodolfo Teófilo<br>ZIP: 60430-160 Fortaleza - Ceará<br>Phone: 85 3366 8252<br>Fax: 85 3366 8316<br>Mobile: 85 9403 1559; 85 8876 4782<br>ivocastelo@uol.com.br
Research Center BVB01<br>Federal University of Roraima<br>Secretary of PROCISA Coordination<br>Room No. 42 Undergraduate Annex/CEDUC - UFRR<br>Av. Ene Garcez, 2413 - Campus Paricarana, Bairro Aeroporto - Boa Vista – RR ZIP: 69.304-000<br>Telephone: 95 3623 5236	Principal Investigator<br>Dr. Allex Jardim da Fonseca<br>Campus Paricarana: Av. Cap. Ene Garcez, nº 2413 Bloco II Bairro: Aeroporto<br>Boa Vista / RR ZIP: 69304-000<br>Telephone: 95 3621 3146<br>allex.j.fonseca@gmail.com
Research Center BSB01<br>Center for Tropical Medicine<br>University of Brasília (UnB)<br>Campus Universitário Darcy Ribeiro, S/N, Asa Norte, Brasília – DF ZIP: 70.904.970<br>Telephones: 61 3107 0085 / 61 3107 0081<br>Email: pgtropical@unb.br	Principal Investigator<br>Dr. Gustavo Romero<br>University of Brasília, Faculty of Medicine, Center for Tropical Medicine.<br>Campus Universitário<br>Asa Norte 70904970 – Brasília, DF – P.O. Box: 04517<br>Telephone: 61 3107 0085<br>Fax: 61 3107 0081<br>romgustavo@gmail.com
Research Center PVH01<br>Center for Research in Tropical Medicine of Rondônia (CEPEM)<br>Av. Guaporé, 127 – Bairro Lagoa<br>ZIP 78918-791<br>Porto Velho, RO<br>Telephone: 69 3216 5442<br>Epidemiology and Clinical Research Group<br>cepemsesauro@gmail.com	Principal Investigator<br>Dr. Dhelio Batista Pereira<br>Clinical Director<br>Center for Research in Tropical Medicine of Rondônia (CEPEM)<br>Av. Guaporé, 127 – Bairro Lagoa<br>ZIP 78918-791<br>Porto Velho, RO<br>Telephone: 69 3216 5442<br>E-mail: dbpfall@gmail.com
Research Center CGB01<br>Júlio Müller University Hospital – Federal University of Mato Grosso	Principal Investigator<br>Dr. Cor Jesus Fernandes Fontes<br>Manager of Teaching and Research

Rua Luiz Philipe Pereira Leite Bairro<br>Alvorada Cuiabá - MT ZIP 78048-902<br>Telephone: 65 3615 7238	Júlio Müller University Hospital/EBSERH<br>Faculty of Medicine<br>Federal University of Mato Grosso<br>Cuiabá-MT<br>Telephone: 65 9981 8777<br>corfontes@gmail.com
Research Center RIO01<br>Evandro Chagas National Institute of Infectious Diseases – Fiocruz<br>Av. Brasil, 4365 - Manguinhos, Rio de Janeiro ZIP: 21040-360<br>Phone: (21) 3865-9595	Principal Investigator<br>Dr. André Siqueira<br>Evandro Chagas National Institute of Infectious Diseases – Fiocruz<br>Av. Brasil, 4365 - Manguinhos, Rio de Janeiro ZIP: 21040-360<br>Phone: (21) 96722-2701
Study Laboratories:	Center for Development and Innovation
	Butantan Institute<br>Avenida Vital Brasil, 1500<br>05503-900 – São Paulo, SP – Brazil<br>Telephone: 11 2627-9833<br>Technical Director:<br>Dr. Vivane Fongaro Botosso<br>Director of the Virology Laboratory<br>Email: vivivane.botosso@butantan.gov.br
Biorepository<br><br>Butantan Institute<br>Av. Vital Brasil, 1500 - São Paulo – SP Brazil – 05503 900<br>Telephone: 11 2627 9300 / Fax: 11 2627 9375	Center for Scientific Development<br>Butantan Institute<br>Avenida Vital Brasil, 1500<br>05503-900 – São Paulo, SP – Brazil<br>Telephone: 11 2627-9580<br>Technical Director:<br>Dr. Sandra Coccuzzo Sampaio Vessoni<br>Director of Scientific Development<br>Email: sandra.coccuzzo@butantan.gov.br<br><br>Technical Director:<br>Dr. Sandra Coccuzzo Sampaio Vessoni<br>Director of Scientific Development<br>Email: sandra.coccuzzo@butantan.gov.br
Funding Sources	Butantan Institute<br>Butantan Foundation<br>UMANE<br>Todos pela Saúde
